# In Vitro Evaluation of *Annona muricata* Leaf Infusion as a Modulator of Antineoplastic Drug-Induced Cytotoxicity in Cancer Cell Lines

**DOI:** 10.3390/ph18081177

**Published:** 2025-08-09

**Authors:** Ariana Cabrera-Licona, Gustavo A. Hernández-Fuentes, Kayim Pineda-Urbina, Alejandra E. Hernández-Rangel, Mario A. Alcalá-Pérez, Janet Diaz-Martinez, Uriel Díaz-Llerenas, José Guzmán-Esquivel, Osval A. Montesinos-López, Juan C. Casarez-Price, Mario Del-Toro-Equihua, Sergio A. Zaizar-Fregoso, Sergio Gamez-Bayardo, Oscar F. Beas-Guzmán, Iván Delgado-Enciso

**Affiliations:** 1 State Cancerology Institute of Colima, Health Services of the Mexican Social Security Institute for Welfare (IMSS-BIENESTAR), Colima 28085, Colima, Mexico; arianacabrera267@gmail.com (A.C.-L.); gahfuentes@gmail.com (G.A.H.-F.); dr.casarezprice@hotmail.com (J.C.C.-P.); 2 Department of Molecular Medicine, School of Medicine, University of Colima, Colima 28040, Colima, Mexico; ahernandez157@ucol.mx (A.E.H.-R.); mequihua@ucol.mx (M.D.-T.-E.); alexzaizar09@gmail.com (S.A.Z.-F.); oscar.beas.11@gmail.com (O.F.B.-G.); 3 Faculty of Chemical Sciences, University of Colima, Coquimatlan 28400, Colima, Mexico; kpineda@ucol.mx; 4 Molecular Medicine Laboratory, Academic Unit of Human Medicine and Health Sciences, Autonomous University of Zacatecas, Zacatecas 98160, Zacatecas, Mexico; marioalcalaperez@uaz.edu.mx (M.A.A.-P.); urieldiazllerenas@gmail.com (U.D.-L.); 5 Research Center in Minority Institutions, Florida International University (FIU-RCMI), Miami, FL 33199, USA; jdimarti@fiu.edu; 6 Department of Dietetics & Nutrition, Robert Stempel College of Public Health & Social Work, Florida International University (FIU-RCMI), Miami, FL 33199, USA; 7 Clinical Epidemiology Research Unit, Mexican Institute of Social Security, Villa de Alvarez, Colima 28984, Colima, Mexico; jose.esquivel@imss.gob.mx; 8 Faculty of Telematics, University of Colima, Colima 28040, Colima, Mexico; oamontes1@ucol.mx; 9 Regional University Hospital, Health Services of the State of Colima, Colima 2805, Colima, Mexico; 10 Posgrado Integral de Biotecnología, Laboratorio de Investigación y Diagnóstico Microbiológico, Universidad Autónoma de Sinaloa, Culiacán 80010, Sinaloa, Mexico; sergiogbayardo@gmail.com

**Keywords:** guanabana, infusion, adjuvant, traditional medicine, folk medicine, antineoplastic drug, breast cancer

## Abstract

**Background/Objectives**: *Annona muricata* (AM), commonly known as soursop or guanabana, has long been used in traditional medicine for its purported anticancer properties. However, scientific studies evaluating its potential enhancing or additive effects with conventional antineoplastic drugs (ADs) remain limited. This study aimed to assess the cytotoxic effects of an aqueous AM infusion alone and in combination with standard ADs in cancer cell lines, while also evaluating its safety in healthy cells. Additionally, we explored the potential molecular interactions of AM metabolites with therapeutic targets using silico modeling. **Methods**: An AM infusion (125 and 250 µg/mL) was tested on two cancer cell lines—MDA-MB-231 (human triple-negative breast cancer) and TC-1 (murine HPV16-positive cancer)—as well as healthy human leukocytes and a non-tumorigenic mouse lung cell line. Cell viability was assessed using the Alamar Blue™ assay. The combined effects of AM with multiple first-line ADs were evaluated. In silico molecular docking was performed with Molegro Virtual Docker to assess the interaction of AM metabolites (quercetin and hyperoside) with the A2B adenosine receptor. Additionally, the physicochemical properties of 13 AD were analyzed to explore correlations with cytotoxic outcomes. **Results**: AM infusion alone exhibited low cytotoxicity in both cancer and healthy cell types. However, when combined with ADs, it enhanced cytotoxic effects in cancer cells while sparing healthy cells at the evaluated concentrations. Docking studies revealed strong interactions between quercetin and hyperoside (major metabolites in the AM infusion) and the A2B receptor, supporting a possible mechanistic explanation for the observed effects. **Conclusions**: AM infusion may act as a chemical modulator, potentiating the effects of conventional ADs in cancer cells while preserving normal cell viability. These findings encourage further preclinical exploration of AM as a complementary agent in integrative oncology.

## 1. Introduction

*Annona muricata* (AM), known in Mexico as guanabana, is a plant species primarily recognized for the commercialization of its fruit, which is used in the food and cosmetic industries [1]. In the past five years, AM has been widely cultivated and distributed throughout Latin America, particularly in Mexico, due to its ease of cultivation and adaptability to various soils and climates [2]. From an ethnomedical perspective, different parts of this plant have been traditionally used for therapeutic purposes. The seeds have been employed for their insecticidal, antimalarial, and antimicrobial effects, while the bark has been used to treat fevers and dysentery. The leaves, on the other hand, have been traditionally utilized for wound treatment, fever, dysentery, diabetes, and malaria, and some studies suggest their potential effect against certain types of cancer [1].

The use of AM leaves has gained special relevance due to their easy availability and minimal impact on the plant [3]. Moreover, since AM is a perennial tree in warm climates, its leaves are present throughout most of the year [3]. Various preparations have been reported, including alcoholic, hydroalcoholic, and aqueous extracts, with infusions being the most commonly used method. Previous phytochemical studies have identified the presence of alkaloids belonging to the isoquinoline, aporphine, and protoberberine families, such as anomurine, anomuricine, cochlaurine, and reticuline [1,4,5,6]. Additionally, acetogenins like annocherimolin, annomontacin, annonacin, querimolin-1, and annomolin have been reported, though these are primarily found in medium- and low-polarity extracts and are less frequently detected in aqueous or alcoholic extractions [7]. Another important family of metabolites found in AM includes flavonoids such as quercetin and kaempferol, along with simple phenols like gallic and tannic acids, which are often found in C-glycosylated and O-glycosylated forms [1,8]. A recent study on AM infusion reported a high presence of phenolic compounds (flavonoids and simple phenols) with an antioxidant activity close to 60%, possibly influenced by these compounds [2].

Several bioactive compounds reported in AM, such as quercetin and kaempferol, have demonstrated cytotoxic and anti-proliferative activities through multiple mechanisms. These include the induction of apoptosis via mitochondrial pathways, the modulation of reactive oxygen species (ROS), the inhibition of PI3K/Akt and MAPK signaling pathways, and cell cycle arrest [9]. Acetogenins like annonacin are known to inhibit mitochondrial complex I, leading to ATP depletion and subsequent cancer cell death [10].

In particular, polar extracts (aqueous and alcoholic) of AM have been found to contain flavonoids, which are natural metabolites that have shown an affinity for specific adenosine receptors [11]. A broad screening of phytochemicals has demonstrated that certain flavone and flavanol derivatives exhibit a relatively high affinity for A3 adenosine receptors, which are implicated in tumor growth, immune modulation, and chemoresistance [12,13]. Structural modifications of these natural metabolites have also been shown to affect other adenosine receptor subtypes within the same family, highlighting their potential importance in cancer cell signaling and therapeutic response [14].

Given the extensive prior research on this species, its high production in Mexico, and the widespread dissemination of its potential applications through media, social networks, and other information channels, AM consumption has expanded across various social sectors as a perceived therapeutic option, particularly for cancer treatment [15,16,17,18,19]. Previous studies conducted by our research group, which examined the perspectives of healthcare professionals and oncology patients on traditional and complementary medicine in western Mexico, revealed that between 30% and 40% of cancer patients consume AM infusions [17,20]. Additionally, a significant proportion of these patients were undergoing active chemotherapy treatment [21].

These findings, along with the ethnomedical background of the species, led us to propose this research with the objective of determining whether AM infusion exerts a cytotoxic effect on cancer cell lines. Furthermore, we aim to evaluate whether, in combination with first-line AD commonly used in the Mexican healthcare system, AM infusion enhances, inhibits, or has no impact on the therapeutic response in these cancer models. This study evaluates its effects on two cancer cell lines (MDA-MB-231 and TC-1), as well as on healthy human leukocytes and a non-tumorigenic murine lung cell line, to assess selectivity and safety. Additionally, considering the phytochemical profile of AM and recent reports of flavonoid enrichment, we employed in silico docking to predict potential interactions with the A2B adenosine receptor, a target implicated in tumor proliferation and immune modulation [12,22]. While A2B is not the most established therapeutic target in TNBC, evidence suggests its involvement in tumor progression and chemoresistance [23,24]. By investigating these effects, we aim to provide a more comprehensive understanding of how traditional plant-based therapies might be integrated into modern cancer treatment regimens.

## 2. Results

### 2.1. AM Infusion in Combination with First Line ADs

The cytotoxic effect of AM infusion alone was first evaluated on the TC-1 and MDA-MB-231 cell lines to determine an appropriate concentration for subsequent combination assays with ADs. It was observed that at 250 µg/mL, the infusion-maintained cell viability ranged from approximately 70% to 97% in both cell lines (Table 1), indicating low intrinsic cytotoxicity. Therefore, the dose of 250 µg/mL was used for all experiments; however, some assays employed lower doses with the purpose of establishing additive or potentiating effects. Concentrations above 350 µg/mL were excluded due to aggregate formation in the culture medium.

**Figure 1:**
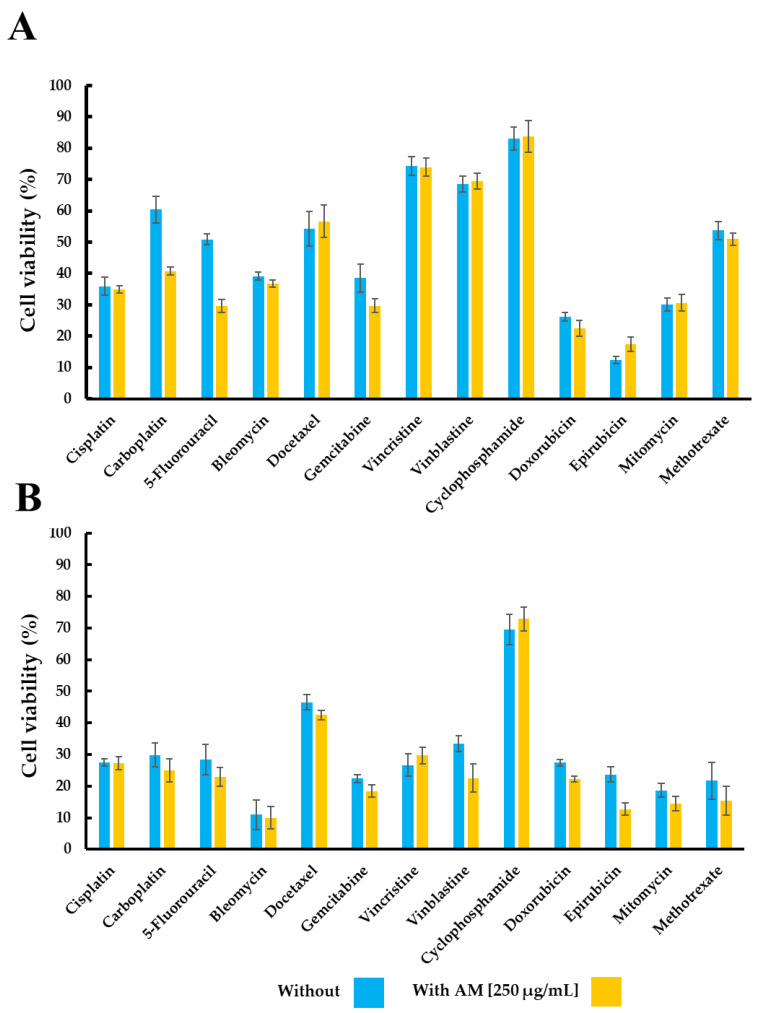
Effect of the AM infusion and ADs on cell viability (%). Panel (**A**) shows the effect in the TC-1 cell line. Panel (**B**) highlights the effects in the MDA-MB-231 cell line. Results are expressed as mean ± SEM from triplicate experiments. “With” refers to treatment with *Annona muricata* leaf infusion at 250 µg/mL along with the AD; “Without” refers to cells treated only with the AD.

Following this, the combined effect of AM infusion with various ADs was assessed. A graphical analysis was performed to illustrate the difference in cell viability between treatments with ADs alone and in combination with AM infusion (Figure 1). Negative values indicate a reduction in viability (i.e., increased cell death or enhanced cytotoxicity) due to the addition of AM, whereas positive values reflect an increase viability (i.e., reduced cytotoxicity). In the TC-1 cell line, the combination of AM infusion with carboplatin and 5-fluorouracil, respectively, resulted in a marked decrease in cell viability, with differences of approximately 20 percentage points compared to treatment with antineoplastic drugs alone. Additionally, epirubicin showed a modest but consistent increase in viability (~5 percentage points), suggesting a potential interaction with the cytotoxicity effect.

Regarding the MDA-MB-231 cell line, the combination of AM infusion with epirubicin and vinblastine resulted in a notable decrease in cell viability, with differences of approximately 10 percentage points compared to antineoplastic drugs alone. Conversely, when cyclophosphamide and vincristine were combined with the infusion, a slight upward trend in cell viability was observed, with marginal differences of approximately 3 percentage points for each one.

Considering the results, these effects appear to depend on both the specific antineoplastic drug and the cell line used. The findings suggest that the interaction between AM infusion and ADs may be compound-specific, potentially enhancing or diminishing cytotoxic effects depending on the drug involved.

### 2.2. AM Infusion in Combination with AD vs. Healthy Cell Lines

To further explore the safety profile of the AM infusion, its effects in combination with selected ADs (5-fluorouracil, carboplatin, epirubicin, and vinblastine) were evaluated in two types of healthy cells: human leukocytes and a non-tumorigenic murine lung cell line (Table 2).

In human leukocytes, treatment with AM infusion alone at concentrations of 125 and 250 µg/mL resulted in a noticeable decrease in cell viability. When combined with ADs, the infusion further amplified cytotoxic effects. Specifically, at 125 µg/mL, 5-fluorouracil and carboplatin reduced leukocyte viability by at least one-third compared to their individual effects, while at 250 µg/mL, the reduction reached up to 50 percentage points. The effects were more pronounced with epirubicin and vinblastine; at 125 µg/mL of AM infusion, leukocyte viability decreased by at least two-thirds relative to treatment with the AD alone, and at 250 µg/mL, viability dropped to approximately 15%.

**Figure 2:**
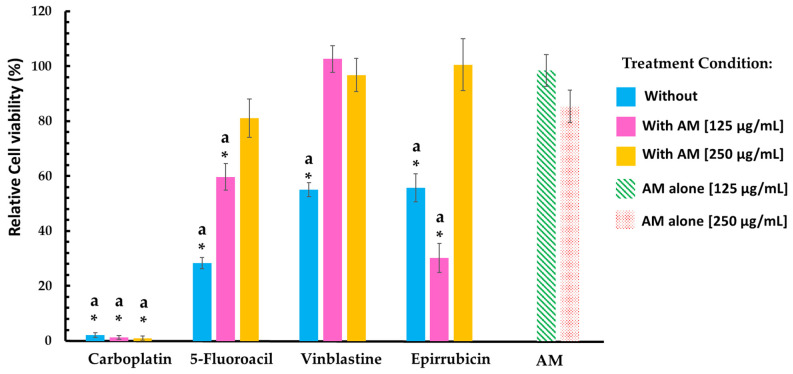
Relative cell viability (%) in non-tumorigenic murine lung cells (TC-1) treated with ADs alone or in combination with the AM infusion. Cells were treated with carboplatin, 5-fluorouracil, vinblastine, and epirubicin either individually (blue bars) or in combination with the AM infusion at 125 µg/mL (pink bars) or 250 µg/mL (yellow bars). Cell viability is expressed as a percentage relative to the untreated control (100%). Results are expressed as mean ± SEM from triplicate independent experiments. Statistical differences were assessed using the Mann–Whitney U test. * *p* < 0.05 vs. AM infusion alone at 250 µg/mL. ^a^ *p* < 0.05 vs. AM infusion alone at 125 µg/mL. “With” refers to treatment with *Annona muricata* leaf infusion at 250 µg/mL along with the antineoplastic drug; “Without” refers to cells treated only with the AD.

In contrast, the AM infusion exhibited a different profile in the healthy mouse lung cell line (Table 2 and Figure 2). The data indicated that AM infusion alone was non-cytotoxic and may even have exerted a protective effect against antineoplastic-induced damage. Co-treatment with AM notably improved viability in cells exposed to 5-fluorouracil, epirubicin, and vinblastine, suggesting a modulatory or antagonistic interaction.

However, in the case of carboplatin, cell viability remained close to 2% across all treatment conditions, including co-treatment with AM infusion. This extreme cytotoxicity prevented the evaluation of any modulatory or protective role of the infusion in this context, indicating that carboplatin alone at the tested concentration was sufficient to induce near-total cell death.

### 2.3. AM Infusion with ADs from an In Silico Point of View

Given the observed differential effects of the AM infusion in both cancerous and healthy cell types, we next aimed to determine whether these effects could be mechanistically supported or correlated with theoretical molecular parameters.

**Figure 3:**
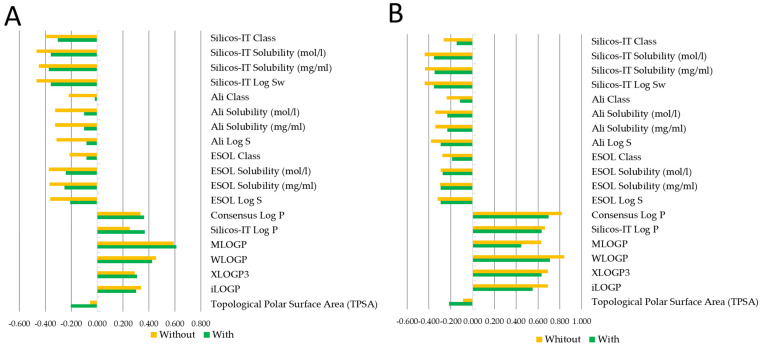
Spearman’s Rho correlation analysis of polar and hydrophobic molecular descriptors in TC-1 and MDA-MB-231 cell lines with and without *Annona muricata* infusion treatment. (**A**) Correlation analysis of polar and hydrophobic molecular descriptors in the TC-1 cell line with (blue) and without (orange) *Annona muricata* infusion treatment. (**B**) Correlation analysis of polar and hydrophobic molecular descriptors in the MDA-MB-231 cell line with (blue) and without (orange) *Annona muricata* infusion treatment. Descriptors include topological polar surface area (TPSA), consensus Log P, Silicos-IT Log P, MLOGP, WLOGP, XLOGP3, and iLOGP. Negative values indicate inverse correlations, while positive values indicate direct correlations.

To this end, molecular descriptors were first calculated for the 13 ADs used in this study (Figure 3). Among the descriptors analyzed, lipophilicity (LogP), molecular weight, topological polar surface area (TPSA), and the number of hydrogen bond donors and acceptors showed the most variation across compounds. Notably, ADs whose cytotoxic effects were modulated by the AM infusion—such as 5-fluorouracil, carboplatin, epirubicin, and vinblastine—tended to share lower LogP values and higher TPSAs, suggesting a potential correlation between hydrophilicity-related properties and the observed modulation by AM infusion.

Taking into account the results obtained in the in vitro section, we also refer to a previously published study by our group that characterized the phytochemical composition of the AM infusion used [2]. This study identified quercetin and glycosylated compounds and analyzed an infusion prepared from the same batch of leaves as used in the present experiments. This approach ensures consistency in the raw material and supports the applicability of the phytochemical findings to the current work. A growing body of evidence suggests that quercetin-type flavonoids may influence cellular signaling pathways involved in drug response. Based on our observations of additive effects in cytotoxicity when AM infusion was co-administered with certain ADs, we propose the mechanistic hypothesis that these flavonoids could modulate membrane permeability, thereby enhancing the intracellular uptake of ADs. However, since the exact bioactive compound (s) responsible for the therapeutic activity and their molecular targets have not yet been experimentally identified, this hypothesis must be interpreted with caution. Further functional assays—such as gene expression analysis, apoptosis assays, reactive oxygen species measurements, and receptor blocking experiments—are necessary to validate this proposed mechanism.

**Figure 4:**
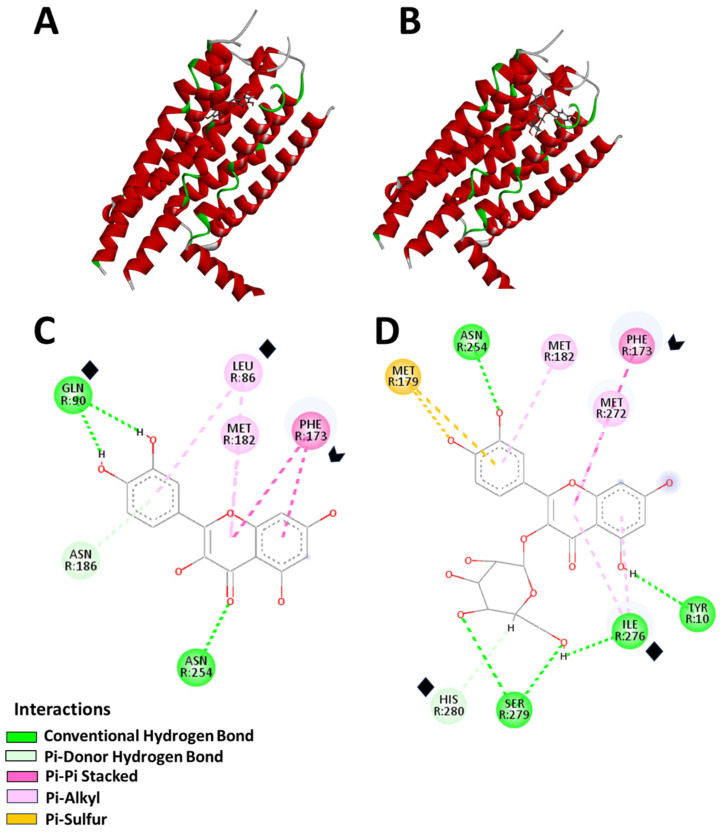
Docking sites and interactions on the adenosine A2B receptor. (**A**) Binding site of quercetin. (**B**) Binding site of hyperoside. (**C**) Residue interactions of quercetin, including hydrogen bonds and π interactions. (**D**) Residue interactions of hyperoside, highlighting the expanded binding site and additional stabilizing interactions due to the glycosidic moiety. Docking simulations were performed using Molegro Virtual Docker 6.0, with identified cavity pockets and a custom search space to enhance accuracy. Binding interactions were categorized based on energy levels, with the MolDock Score used to rank ligand binding affinity. Strong interactions are observed with PHE 173 for both ligands (arrowhead). Mild interactions are noted with GLN 90, LEU 86, and TYR 10 for quercetin, and HIS 280 and ILE 276 for hyperoside (♦). Weak interactions are seen with ASN 186, ASN 254, and MET 182 for quercetin, and ASN 254, MET 182, MET 272, and SER 279 for hyperoside.

Given the more pronounced effects observed in the MDA-MB-231 cell line and the possibility that AM infusion enhances membrane permeability—thereby facilitating increased AD uptake—the docking simulation focused on the A2B adenosine receptor, a membrane protein implicated in tumor growth, immune modulation, and chemoresistance [25,26] (Figure 4).

Both quercetin and hyperoside successfully bound to the A2B receptor in distinct binding poses (Figure 4), with their most favorable configurations shown in Figure 4A,B. The MolDock Scores were 82.00 a.u. for quercetin and 104.20 a.u. for hyperoside, indicating strong binding affinities. Hydrogen bonding and π-electron interactions were the dominant forces stabilizing ligand–receptor complexes. Quercetin formed hydrogen bonds with GLN 90 (−13.30 a.u.), ASN 254 (−5.36 a.u.), and ASN 186 (−7.79 a.u.), along with π-electron interactions involving PHE 173 (−22.08 a.u.), LEU 86 (−16.25 a.u.), and MET 182 (−7.32 a.u.) (Figure 4C,D). Hyperoside, due to its glycosidic moiety, exhibited a broader interaction profile, forming hydrogen bonds with TYR 10 (−10.60 a.u.), ILE 276 (−19.35 a.u.), SER 279 (−6.33 a.u.), ASN 254 (−9.39 a.u.), and HIS 280 (−11.97 a.u.), along with π-electron interactions with PHE 173 (−27.47 a.u.), MET 182 (−5.95 a.u.), MET 272 (−9.31 a.u.), and MET 179 (0.34 a.u.).

Notably, both ligands consistently interacted with the PHE 173 residue, highlighting its pivotal role in stabilizing ligand binding. The docking simulations revealed strong binding energies of −122.5 kcal/mol for quercetin and −117.3 kcal/mol for hyperoside, further supporting their high affinity for the receptor’s active site.

These results showed that quercetin-type flavonoids may influence the cellular signaling pathways involved in drug response, providing a mechanistic rationale for the observed enhancement or modulation of cytotoxicity when AM infusion was co-administered with specific ADs in cancer cells. Similar studies have been performed, with references demonstrating that similar methodological approaches have been successfully applied in studies involving other plant extracts [27]. However, these in silico findings provide preliminary mechanistic insight consistent with our hypothesis, although experimental validation remains essential.

## 3. Discussion

In this study, the effect of AM infusion on cell viability was evaluated in vitro using two cancer cell lines (TC-1 and MDA-MB-231), as well as healthy cells represented by human leukocytes and mouse lung epithelial cells. The results provide important insights regarding the biological activity of this herbal remedy, commonly used in traditional medicine.

Firstly, under experimental conditions, the AM infusion did not exhibit significant cytotoxic effects in either cell line, especially when compared to other plant extracts that typically show more pronounced effects at similar concentrations [28]. However, interesting trends emerged when the infusion was combined with selected ADs. In this context, the ADs were tested at concentrations simulating plasma levels, meaning that the evaluated doses corresponded to those expected in the bloodstream of an average patient [29]. These agents demonstrated notable cytotoxic effects, reducing cell viability to approximately 50% on average, with some cases maintaining viability as high as 60%. When combined with AM infusion, some ADs exhibited enhanced cytotoxicity, while others showed a reduction in their cytotoxic effects, suggesting a potential modulatory role of the infusion.

This effect may be linked to the metabolites present in the AM infusion, particularly flavonoids, which were identified in high abundance (including quercetin, glycosylated derivatives, and other flavonoids) [1,30]. Particularly, flavonoids have been shown to modulate the efficacy of ADs through various mechanisms. One key mechanism involves inhibiting efflux pumps like P-glycoprotein (P-gp), thereby increasing the intracellular retention of ADs [31]. This phenomenon has been reported in several cancer cell lines, including colon and pancreatic cancers [31,32].

Additionally, flavonoids can alter membrane fluidity, potentially affecting drug permeability [33]. Their impact depends on their chemical nature: lipophilic flavonoids interact with the hydrophobic regions of the membrane, increasing rigidity and stabilizing the bilayer, whereas hydrophilic flavonoids interact with polar head groups, enhancing bilayer fluidity [34]. These changes can modulate membrane permeability and influence the activity of transport proteins, such as ATP-binding cassette (ABC) transporters [33]. Beyond their role in drug transport, flavonoids may also enhance antineoplastic-induced cytotoxicity by modulating oxidative stress and apoptosis. By increasing reactive oxygen species (ROS) production, they can sensitize cancer cells to oxidative damage [35]. Furthermore, flavonoids can influence apoptosis by activating pro-apoptotic factors such as Bax or downregulating anti-apoptotic proteins like Bcl-2 [31]. These mechanisms could help explain the differential effects observed in the cancer cell lines studied. While some ADs exhibited increased cytotoxicity in combination with the infusion, others showed reduced effectiveness. Other antioxidant phytochemicals, including phenols and flavonoids structurally similar to those in AM, have shown anticancer activity through mechanisms such as ROS modulation, apoptosis induction, and the inhibition of the PI3K/Akt and MAPK pathways [36,37]. In addition, some natural extracts and isolated compounds (quercetin) have also enhanced antineoplastic efficacy via molecular synergy [38,39], supporting the potential of AM infusion to act through similar pathways, supporting the results obtained in this investigation. However, further experiments are required to confirm these hypotheses and clarify the precise interactions between AM metabolites and ADs.

An additional consideration involves the interaction-specific effects observed between AM infusion and selected ADs such as epirubicin, vinblastine, bleomycin, and carboplatin. Although these agents differ mechanistically—targeting topoisomerases, the mitotic spindle, or directly damaging DNA—their distinct physicochemical properties may influence how AM infusion modulates their cytotoxicity. For instance, 5-fluorouracil and carboplatin, which primarily act on nuclear DNA, have relatively lower molecular weights and are more hydrophilic, potentially limiting their intracellular accumulation [40]. This is supported by their lower LogP values and higher topological polar surface areas (TPSAs), which correlate with reduced membrane permeability and passive diffusion. In contrast, epirubicin and vinblastine, being more lipophilic, may be more readily internalized when membrane fluidity is altered, a condition favored by the presence of certain flavonoids like quercetin in the AM infusion. These flavonoids can also modulate the activity of efflux transporters, potentially enhancing the intracellular retention of these ADs [31,41,42]. Thus, the interaction between AM infusion and ADs may depend not only on the drugs’ mechanisms of action but also on physicochemical features that govern their cellular uptake and distribution. These findings reinforce the need for further functional and pharmacokinetic studies to elucidate these interactions [29].

In this study, the evaluation of AM infusion effects on two healthy cell lines showed distinct responses. In leukocytes, the infusion exhibited significant cytotoxicity, which may be related to their limited protective mechanisms compared to cancer cells that have developed advanced defenses against cytotoxic agents [43,44]. While leukocytes are healthy, non-transformed cells with limited protective adaptations, cancer cells develop advanced defense strategies that enhance their survival under stress, including exposure to phytochemicals. Interestingly, this finding also opens the possibility of exploring new mechanisms and effects on different cell lines, such as leukemia models, where the cytotoxic impact of AM infusion could be further investigated [44,45,46].

Although the observed protective effect of AM infusion on murine lung epithelial cells suggests potential tissue-specific benefits, the underlying mechanisms remain unclear. It is plausible that differential expressions or activities of drug transporters (e.g., ABC transporters) and antioxidant defenses contribute to this selective protection [47]. Lung epithelial cells may exhibit higher basal antioxidant capacity or more efficient detoxification pathways compared to leukocytes, which are known to be more vulnerable to oxidative stress and toxic insults [48]. Given that ABC transporters are generally more abundant in leukocytes, it is possible that the pronounced cytotoxicity observed in these cells results from the inhibition or blockade of these transporters by compounds in AM infusion or from membrane alterations that increase the intracellular accumulation of ADs. Conversely, the higher antioxidant capacity of lung epithelial cells may confer resistance and protect them from such cytotoxic effects. While this hypothesis provides a plausible explanation for the differential responses, further studies are required to confirm these mechanisms.

Furthermore, it is important to highlight that, in ethnomedicinal contexts, AM infusions have traditionally been regarded as both safe and effective. A key consideration regarding the bioactive metabolites is the concentration tested in vitro (250 µg/mL). Based on the average yield of approximately 200 mg of extract per infusion preparation (as described in the preparation section), a 70 kg individual would need to consume around 1.25 L of infusion in a single dose to reach comparable concentrations [49]. However, traditional use typically involves smaller, distributed doses throughout the day, rather than a single concentrated intake. Moreover, due to hepatic metabolism and enzymatic degradation in the gastrointestinal tract, only a fraction of these compounds is expected to reach systemic circulation, making such concentrations unlikely under normal physiological conditions [50,51]. Therefore, while our findings provide valuable insight into potential cellular effects, they must be interpreted with caution, as in vitro conditions may not fully reflect in vivo scenarios. These observations underscore the need for further pharmacokinetic and formulation studies to determine whether therapeutic levels of bioactive metabolites can be achieved through conventional or optimized delivery strategies. Additionally, the differential response observed in healthy murine lung cells—where AM infusion enhanced viability with some drugs and decreased it with others—highlights the relevance of cell type and drug mechanism in modulating its effects.

Experimentally, in MDA-MB-231 cells, the combination of AM infusion with vinblastine, bleomycin, 5-fluorouracil, and carboplatin led to increased cytotoxicity. These effects may be attributed to the presence of phenolic compounds—both simple phenols and polyphenols—in the infusion, which have been shown in in vitro models to promote apoptosis by increasing TGF-β and extracellular adenosine levels while inhibiting VEGF expression [52,53]. Together with the results from lipophilicity descriptor analyses, these findings support the potential role of AM infusion in enhancing the efficacy of chemotherapy. However, further in vitro and in vivo studies are needed to validate these observations and clarify the underlying mechanisms.

In order to propose a possible hypothesis to explain the results observed, particularly in the MDA-MB-231 cell line, in silico experiments were conducted to evaluate the interaction of previously identified metabolites in the infusion [2] with the A2B adenosine receptor, a membrane protein known to be expressed in this cell line. The in silico analysis suggests that AM infusion may enhance the cytotoxicity of specific ADs through two potential mechanisms. Molecular docking studies targeting the A2B adenosine receptor revealed that quercetin and hyperoside—both present in the infusion—establish strong hydrogen bonds and hydrophobic interactions with this receptor [54,55], potentially modulating cellular sensitivity to treatment. While our previous phytochemical analyses confirmed the presence of quercetin and hyperoside in the leaf’s infusion—compounds with documented anticancer activities [56,57]—we recognize that AM infusions are chemically complex.

Recent studies have used similar models combining in vitro and in silico approaches to evaluate plant-derived compounds in breast cancer, including MDA-MB-231 cells [27,58], supporting the relevance and methodology of the present work. However, it cannot be ruled out that other molecular pathways, additional metabolites, or the combined effect of multiple components within the infusion may also contribute to the observed activity. This represents an initial step toward understanding the molecular basis of the interaction and supports further investigation. Future studies should employ advanced phytochemical techniques (e.g., HPLC-MS, bioassay-guided fractionation) to isolate and characterize novel or understudied constituents, which may offer unique mechanisms of action and greater translational novelty [27,58].

While the results obtained are promising, certain limitations must be considered. Establishing marker metabolites would also aid in standardization. One key aspect is the need for a more detailed phytochemical analysis of AM infusion. Although previous studies indicate that its infusion is rich in flavonoids, simple phenols, and alkaloids, it is essential to identify and quantify these compounds, as well as assess their variability across different regions, climates, and soil conditions [59,60]. Another important limitation of this study is the use of only two cancer cell lines, which may restrict the generalizability of the findings. Future research should include additional cancer cell lines, such as other hormone receptor-positive breast cancer models and diverse tumor types, to comprehensively evaluate the effects of AM infusion across a broader spectrum of cancers [21]. In addition, in vivo studies are necessary to confirm the cytotoxic and modulatory effects observed in vitro and to better understand the systemic implications of AM infusion in a physiological environment. Finally, although formal synergy analysis was not performed, the observed enhancement in cytotoxicity when combining AM infusion with several ADs suggests potential enhancing or additive effects. According to Wagner (2011), such interactions can be preliminarily inferred when the combined effect exceeds that of the individual components [61].

However, while the use of AM infusion in combination with ADs shows potential, careful evaluation is necessary. Reducing chemotherapy dosages through plant-based compounds could be beneficial, but there is also a risk of unintended effects on healthy cells, which may exacerbate chemotherapy-related toxicity or introduce new complications. Therefore, further studies (in vivo and in vitro) are required to establish the safety profile of these combinations, define appropriate dosage thresholds, and ensure their integration into cancer treatment remains both effective and safe. Understanding the impact of natural compounds on chemotherapy efficacy in a controlled setting is crucial for advancing their potential role in oncology research.

## 4. Materials and Methods

### 4.1. AM Infusion Preparation

*Annona muricata* L. leaves were collected from the Comala Center (19.333692, −103.761888). This species, widely distributed in the region, was identified by comparison with a registered herbarium specimen (MEXU:981827) [62]. Leaves were dried at 30 °C for 72 h. Following traditional herbalist methods, an infusion was prepared by adding 7 leaves (1 g each) to 1 L of boiling water (98 °C), steeping for 15 min, and filtering. In the laboratory, these conditions were replicated, and the infusion was lyophilized for concentration. The final product was stored in amber glass jars under an inert atmosphere at −20 °C until use [63].

It is important to highlight that the phytochemical composition of the AM infusion used in this study has been previously characterized by our group [2]. The infusion was prepared from the same batch of leaves collected from the same location and processed using identical methods as those described in the earlier published work [2]. This prior study identified key bioactive compounds, including quercetin, glycosylated flavonoids, anthrone-like structures, and phenolic acids such as gallic acid. The consistency in raw material and preparation supports the relevance and applicability of the phytochemical profile to the current experiments.

### 4.2. Reagents and Treatments

Various ADs were utilized in this study and prepared under standard conditions with the diluents recommended by the manufacturers. Stock solutions were stored according to the conditions specified by the manufacturers. The agents, listed with their respective commercial presentations as acquired, included epirubicin hydrochloride (Papluf, 2 mg/mL, ULSA TECH, Mexico City, Mexico), docetaxel (Daxel, 20 mg/mL, RTU, Mexico City, Mexico), cisplatin (ACCOCIT, 1 mg/mL, Accord, Ahmedabad, India), gemcitabine hydrochloride (Enekamub, 1 g/25 mL, Glenmark, Mumbai, India), 5-fluorouracil (Ulsacil, 250 mg/10 mL, ULSA TECH, Mexico City, Mexico), mitomycin (Kenomix, 5 mg/10 mL, Kemex, Mexico City, Mexico), methotrexate (Traxacord, 500 mg/5 mL, Accord, Ahmedabad, India), cyclophosphamide monohydrate (Mexciken, 200 mg/10 mL, Kemex, Mexico City, Mexico), vinblastine sulfate (Diranovyl, 10 mg/10 mL, ULSA TECH, Mexico City, Mexico), bleomycin sulfate (Novamexan, 9.9 mg/10 mL, Kemex, Mexico City, Mexico), carboplatin (Nuvaplast, 150 mg/15 mL, Accord, Ahmedabad, India), vincristine sulfate (VinLon, 1 mg/mL, Celon Labs, Hyderabad, India), and doxorubicin hydrochloride (50 mg/25 mL, TEVA, Petah Tikva, Israel). These concentrations correspond to the commercial presentations at which each AD was acquired. The evaluated concentrations correspond to the reported IC_50_ values: bleomycin (87.9 µM), vinblastine (0.035 µM), cyclophosphamide (139.7 µM), carboplatin (13.5 µM), methotrexate (3.46 µM), mitomycin (2.18 µM), 5-FU (153.4 µM), vincristine (0.1 µM), gemcitabine (89.3 µM), docetaxel (5.74 µM), epirubicin (16.6 µM), doxorubicin (6.73 µM), and cisplatin (58.32 µM).

### 4.3. In Vitro Evaluation

TC-1 cells (ATCC: CRL-2493) were selected for their expression of HPV16 E6 and E7 oncogenes, making them relevant for evaluating immunomodulatory effects. MDA-MB-231 cells (ATCC: HTB-26TM) represent human triple-negative breast cancer. Both cell lines were cultured in DMEM/F-12 (Biowest) supplemented with 10% FBS and 1× penicillin/streptomycin (Gibco) at 37 °C in a 5% CO_2_ humidified incubator. Subculturing was performed using 0.25% Trypsin-EDTA (Gibco, Thermo Fisher Scientific, Waltham, MA, USA). Cells were seeded at a density of 1 × 10^4^ per well in 96-well plates (Corning^®^, Corning Inc., Corning, NY, USA) and incubated for 24 h before replacing the medium with the treatments. After 48 h of treatment, cells were incubated with 1× Alamar Blue™ reagent (Sigma-Aldrich, Merck KGaA, Darmstadt, Germany) in a serum-free medium for 4 h. This assay was chosen over traditional MTT due to its relative insensitivity to precipitation artifacts that may occur with crude plant extracts and its similar sensitivity and reproducibility in cytotoxic screening [64,65]. Optical density was measured at 570–600 nm using a microplate reader (iMark, Bio-Rad, Laboratories, Hercules, CA, USA). Cytotoxicity was calculated using the following formula: 100 − [(experimental OD − blank OD)/(control OD − blank OD) × 100]. IC_50_ values were determined using nonlinear regression curve analysis [63].

#### 4.3.1. Isolation of Lymphocytes from Human Peripheral Blood and Cell Viability Test

The isolation of lymphocytes from human peripheral blood and the assessment of cell viability were conducted following the methods described by [66], with minor modifications. Heparinized venous blood samples (10 mL) were collected in September from three healthy male volunteers aged 22–25 years who met the inclusion criteria of good general health, absence of chronic or active infectious diseases, and having fasted for at least 8 h before collection. Participants had not used any medications or drugs in the 72 h preceding the study and were selected based on normal hematological parameters confirmed by a complete blood count (CBC). Exclusion criteria included regular alcohol or tobacco consumption, recent tattoos within the last six months, active infections, medical conditions affecting hematological parameters, significant CBC abnormalities, or participation in other clinical or experimental studies. All participants provided oral informed consent.

Blood collection took place at the State Cancerology Institute of Colima, Health Services of the Mexican Social Security Institute for Welfare (IMSS-BIENESTAR). The samples were centrifuged at 2500 rpm for 20 min to separate the cellular layer, which was diluted 1:1 with HBSS, layered over Ficoll-Paque, and centrifuged at 1500 rpm for 10 min. The isolated lymphocytes were washed twice in RPMI 1640 medium by centrifugation at 1500 rpm (201× *g*) for 10 min and resuspended in RPMI 1640 medium (37 °C) supplemented with 1% penicillin/streptomycin. Cell viability was immediately assessed using a Neubauer chamber and the trypan blue exclusion method. A mixture of 10 μL of the cell pellet and 10 μL of trypan blue was incubated for 3 min, and the proportion of dead cells was determined by counting 100 consecutive cells in duplicate. Viability was evaluated both before and after treatment.

The evaluation included the ADs 5-fluorouracil (75 μM), carboplatin (67.5 μM), epirubicin (7.5 μM), and vinblastine (0.0351 μM), chosen for their distinct effects when combined with AM infusion, which was tested at 125 and 250 μg/mL. All experiments were conducted in triplicate as independent assays [66]. The study protocol was approved by the Regional University Hospital of the Health Services of the State of Colima (Reference Number: CEI2024/1/CR/EXP/FAR/185, 9 May 2024).

#### 4.3.2. Establishment of Primary Culture of Mouse Lung Fibroblasts

The lung from a BALB/C (nu/nu) nude mouse was carefully excised under sterile conditions and following the ethical principles of working with animals [67]. Then it was washed with cold 1× PBS sterile, and fragments of approximately 1 cm^2^ were obtained and maintained in 1.5 mL microtubes with DMEM/F-12 medium (Biowest^®^, Bradenton, FL, USA) with 1× penicillin/streptomycin/amphotericin B (Antibiotic-Antimycotic 100×, Biowest^®^, Bradenton, FL, USA). Subsequently, a sub-fragmentation was performed, and the tubes were centrifuged at 1200 rpm for 5 min; the supernatants were recovered and incubated with 500 μL of 0.025% trypsin-EDTA solution (GibcoTM, Life Technologies Corp, NY, USA) at 37 °C for 10 min with shaking at 1000 rpm (ThermoMixer^®^, Eppendorf, Hamburg, Germany). Trypsin was inactivated with DMEM/F-12 medium supplemented with 30% fetal bovine serum (Antibiotic-Antimycotic 100X, Biowest^®^, Bradenton, FL, USA), and the tubes were centrifuged under the same conditions. Cell pellets were recovered, resuspended in DMEM/F-12 medium with 30% serum, seeded in TC-treated 24-well flat-bottom plates (TPP^®^, Techno Plastic Products AG, Trasadingen, Canton of Schaffhausen, Switzerland), and incubated in a humidified atmosphere with 95% air and 5% CO_2_ at 37 °C (ESCO CelCulture^®^ Incubator, Hatboro, PA, USA) for 24 h. After that, the medium was replaced to remove unattached cells and the culture was maintained until 70–80% confluence with DMEM/F-12 medium with 20% serum. The cells were subcultured with trypsin and transferred to a 25 cm^2^ tissue culture flask with a filter screw cap PTFE membrane with pores 0.22 μm in size (TPP^®^, Techno Plastic Products AG, Trasadingen, Canton of Schaffhausen, Switzerland). When the cells showed a fibroblastoid morphology in pass two, the cells were used in viability assays.

### 4.4. In Silico Analysis

Theoretical analysis of 13 ADs was performed using the SwissADME platform, which calculated 41 molecular descriptors to evaluate the physicochemical, pharmacokinetic, and drug-likeness properties. Physicochemical properties included molecular weight, number of heavy atoms, fraction of sp^3^ carbons, number of rotatable bonds, hydrogen bond acceptors and donors, molar refractivity, and topological polar surface area. Lipophilicity was assessed through various descriptors, including iLOGP, XLOGP3, and consensus Log P. Water solubility was evaluated using parameters such as ESOL Log S and Silicos-IT Log S. Pharmacokinetic parameters covered gastrointestinal absorption, blood–brain barrier permeability, P-glycoprotein substrate status, and cytochrome P450 enzyme inhibition potential (CYP1A2, CYP2C19, CYP2C9, CYP2D6, and CYP3A4). Drug-likeness was evaluated using Lipinski, Ghose, Veber, Egan, and Muegge criteria, along with bioavailability score, PAINS alerts, Brenk structural alerts, lead-likeness, and synthetic accessibility [68,69].

### 4.5. Molecular Docking

Molecular docking of quercetin and its O-glycosidic derivative (hyperoside) with the human adenosine A2B receptor was performed using Molegro Virtual Docker 6.0 software. The target protein structure (8HDO) was obtained from the Protein Data Bank [25,26]. Quercetin and hyperoside were selected for docking due to their abundance in the AM infusion [1]. The MDA-MB-231 breast cancer cell line was chosen as it expresses high levels of A2B adenosine receptors. Furthermore, the potential of the A2B receptor in modulation under hypoxic conditions—a hallmark of the tumor microenvironment in TNBC—to shift signaling towards a pro-tumorigenic phenotype shown in recent studies suggests that targeting adenosine signaling pathways may influence therapeutic responsiveness in TNBC models. More recent studies suggest that targeting adenosine signaling pathways may influence therapeutic responsiveness in TNBC models [12,22]. Docking simulations were conducted with 20 runs per ligand, using the MolDock Score for scoring. The custom search space was defined around a cavity pocket identified in the reported structure. Top docking poses were analyzed, and binding interactions were categorized by energy levels: main (<−20 a.u.), strong (−20 to −10 a.u.), intermediate (−10 to −5 a.u.), or weak (−5 to 0 a.u.). Docking validation included comparing ligand affinities and predicted binding sites with the amino acid residues of 8HDO.

### 4.6. Statistical Analysis

Results are expressed as mean ± standard error of the mean (SEM) or standard error of the mean (SEM). For the cell viability analysis, results were averaged over three independent experiments with seven replicates per experiment. Differences between groups were assessed using the Mann–Whitney U test [70]. Outliers were identified and removed using Grubb’s test at a significance level of α = 0.05, to ensure the validity of the data. This analysis was applied to each treatment group individually. For the analysis of the correlation between physicochemical descriptors, the Spearman correlation was employed. Data was analyzed using SPSS Statistics version 20 software (IBM Corp., Armonk, NY, USA) [71].

## 5. Conclusions

This study provides evidence that AM infusion alone does not exhibit significant cytotoxicity in cancer cell lines; however, its combination with specific ADs influences the efficacy of the compounds, either enhancing or inhibiting their cytotoxic effects. These interactions may be attributed to the presence of flavonoids and other bioactive compounds, which could modulate drug transport, oxidative stress, and apoptosis pathways. The in silico analysis further supports the potential involvement of key metabolites in these mechanisms. While these findings highlight the relevance of exploring plant-based adjuvants in cancer treatment, further research is needed to assess the consistency of this profile across different environmental conditions and batches, as well as to evaluate the safety, pharmacokinetics, and clinical applicability of AM infusion as a potential adjuvant in cancer therapy.

## Figures and Tables

**Table 1:** Cell viability in TC-1 and MDA-MB-231 cells following treatment with antineoplastic drugs or without *Annona muricata* leaf infusion.

Treatment	Cell Viability (%)			
	TC-1		MDA-MB-231	
	Without	With	Without	With
*Annona muricata* infusion [250 µg/mL]	97.51 ± 2.53		70.27 ± 10.41	
Cisplatin	35.95 ± 2.98	34.92 ± 1.26	27.48 ± 1.06	27.20 ± 2.08
Carboplatin	60.45 ± 7.23	40.76 ± 1.34	29.83 ± 3.80	25.03 ± 3.65
5-Fluoracil	50.88 ± 1.69	29.67 ± 2.09	28.43 ± 4.87	22.93 ± 2.87
Bleomycin	39.12 ± 1.28	36.76 ± 1.05	10.94 ± 4.68	9.99 ± 3.50
Docetaxel	54.27 ± 5.55	56.69 ± 5.25	46.51 ± 2.37	42.47 ± 1.47
Gemcitabine	38.56 ± 4.49	29.73 ± 2.26	22.38 ± 1.21	18.40 ± 1.90
Vincristine	74.34 ± 3.05	73.97 ± 2.87	26.69 ± 3.47	29.73 ± 2.66
Vinblastine	68.55 ± 2.58	69.40 ± 2.55	33.42 ± 2.56	22.57 ± 4.54
Cyclophosphamide	83.10 ± 3.74	83.76 ± 5.15	69.58 ± 4.79	72.91 ± 3.80
Doxorubicin	26.15 ± 1.44	22.48 ± 2.61	27.38 ± 1.06	22.27 ± 0.84
Epirubicin	12.28 ± 1.16	17.18 ± 2.32	23.72 ± 2.34	12.69 ± 1.94
Mitomycin	30.15 ± 2.04	30.66 ± 2.54	18.69 ± 2.13	14.46 ± 2.20
Methotrexate	53.77 ± 2.84	51.00 ± 1.98	21.69 ± 5.90	15.49 ± 4.57

“With” refers to treatment with *Annona muricata* leaves infusion at 250 µg/mL along with the AD; “Without” refers to cells treated only with the AD. Results are expressed as mean ± SEM from triplicate experiments.

**Table 2:** Cell viability in non-tumorigenic murine lung cell line with ADs and AM infusion.

Treatment	Cell Viability (%)		
	Without	With AM Infusion	
		125 μg/mL	250 μg/mL
Leukocytes			
*Annona muricata* infusion		61.34 ± 9.54	31.81 ± 6.61
5-Fluoracil	90.37 ± 5.48	62.39 ± 9.80	46.18 ± 12.35
Carboplatin	84.29 ± 4.42	68.49 ± 6.14	42.13 ± 7.32
Epirubicin	86.51 ± 1.80	28.28 ± 5.97	18.54 ± 3.62
Vinblastine	90.44 ± 1.96	30.91 ± 9.46	15.58 ± 4.47
Murine Lung Cell Line			
*Annona muricata* infusion		98.42 ± 5.83	85.37 ± 5.88
5-Fluoracil	28.40 ± 2.03	59.72 ± 4.87	81.04 ± 6.98
Carboplatin	2.22 ± 0.82	1.38 ± 0.75	1.22 ± 0.80
Epirubicin	55.78 ± 5.10	90.24 ± 5.83	100.5 ± 9.42
Vinblastine	55.07 ± 2.60	102.6 ± 4.81	96.77 ± 5.88

Evaluation time: 24 h. Cell count: 5000 cells per well. Experiments were performed independently in triplicate. Plasma concentrations reported for patients were used for the following drugs: 5-fluorouracil (75 μM), carboplatin (67.5 μM), epirubicin (7.5 μM), and vinblastine (0.0351 μM). “With” refers to treatment with *Annona muricata* infusion at 250 µg/mL along with the AD; “Without” refers to cells treated only with the antineoplastic drug (AD). Results are expressed as mean ± SEM.

## Data Availability

The datasets used and/or analyzed during the current study are available from the corresponding author upon reasonable request.
